# Patient-reported orofacial-dental pain severity and tele-triage decisions during COVID-19 pandemic: Does the severity of pain drive tele-triage decisions?

**DOI:** 10.1186/s12903-022-02340-w

**Published:** 2022-07-27

**Authors:** Shaymaa Abdulreda Ali, Walid El Ansari

**Affiliations:** 1 Unit of Orthodontics, Hamad Dental Center, Hamad Medical Corporation, Doha, Qatar; 2 Department of Surgery, Hamad General Hospital, Hamad Medical Corporation, Doha, Qatar; 3 College of Medicine, Qatar University, Doha, Qatar; 4 Weill Cornell Medicine – Qatar, Doha, Qatar

**Keywords:** Triage, Hotline, Telemedicine, Orofacial pain, COVID-19

## Abstract

**Background:**

Globally, with the COVID-19 pandemic, dental services were limited to emergency/ urgent conditions and were provided only after tele-triage referral for face-to-face management. However, no previous research explored whether the pain severity (PS) drives the tele-triage decisions. The current study examined the association between PS and tele-triage decision of whether to manage the condition remotely or refer the caller for face-to-face management.

**Methods:**

This retrospective cross-sectional study analyzed the PS reported by hotline callers, using numerical rating scale (NRS-11), during the first wave of COVID-19 lockdown (23 March–31 August 2020) and its association with tele-triage decision controlling for age, sex, history of chronic illness, and dental discipline needed. Binomial logistic regression assessed the association between the PS (exposure) and tele-triage decision (outcome). ANOVA compared PS across tele-triage categories, dental history and tentative diagnosis.

**Results:**

PS was significantly associated with tele-triage decisions (*p* < 0.05). An increase in pain score by 1 unit was associated with 1.4 times increased odds of face-face referral (95% CI: 1.26–1.54). Pediatric/ adolescent patients (9–18 years) (odds ratio (OR) = 2.07; 95% CI: 1.07–4.02), history of chronic illness (OR = 2.12; 95% CI:1.28–3.51), need for surgical specialty (OR = 1.93; 95% CI: 1.22–3.04) and orthodontic specialty (OR = 7.02; 95% CI: 3.54–13.87) were independently associated with tele-triage decision. PS was highest for the emergency triage category (8.00 ± 2.83, *P* < 0.0001), dental history of tooth with cavity or filling (6.65 ± 2.024, *P* < 0.0001), and the tentative tele-diagnosis of cellulitis (7.75 ± 2.872, *P* < 0.0001).

**Conclusions:**

During COVID-19 pandemic, tele-triage decisions were significantly influenced by patient-reported PS, adjusting for a range of variables. Despite this, referral for face-to-face management was individualized and driven by the tripartite considerations of the reported pain, clinical judgement, and the high transmission characteristics of COVID-19.

**Supplementary Information:**

The online version contains supplementary material available at 10.1186/s12903-022-02340-w.

## Background

As COVID-19 affected the world, it was soon classified as a pandemic by the World Health Organization [1], and nationwide complete lockdown became the sole option [2]. This affected all aspects of healthcare care, including oral health. A complete move from the traditional care became critical, and virtual health technologies came to the forefront out of necessity to continue patient care and reduce the risk of transmission [3]. In line with recommendations of the World Health Organization [4], Hamad Dental Center (HDC), the sole public tertiary dental services provider in Qatar, initiated changes related to all aspects of dental services. These included patient triage for COVID-19 symptoms, operatory preparation, infection control, and selection of low-risk interventions. HDC also initiated a dedicated teledentistry (TD) hotline to ensure the continuity of care to patients including triage, consultation, identification of oral diseases, and treatment and/or referral according to the urgency of oral/dental condition [5]. TD applies to the remote provision of dental care without physical contact using a variety of technologies [6], including synchronous real-time communication like audioconferencing, videoconferencing, instant messaging communication software [7], or asynchronous store-and-forward communication. Tele-triage applies to the use of TD to interview callers, assess urgency and sort patients by priority and level of care required [8, 9]. The aim was to maintain the continuity of services and to avoid missing serious or emergency dental conditions.

As part of the COVID-19 measures, the American Dental Association (ADA) published guidelines with definitions for different categories of dental conditions [6]. Dental pain was a fundamental component in these definitions as it is a highly prevalent and significant health problem that reduces quality of life and well-being [10–13].

Pre-pandemic literature suggested that patients seek emergency appointments in dental practices, and to a certain extent in general medical practices and emergency departments because of ease of access, anxiety from dental intervention, or the notion that a course of antibiotics will treat the condition [14]. COVID-19 pandemic influenced the utilization of emergency dental services [15], and its impact on patterns of dental emergencies was evident in the increased prevalence of dental pain compared to other oral health related problems [16–21]. It has also been suggested that during the pandemic, only patients with acute pain remained constant [22], while the less acute but nevertheless existing dental problems were neglected [23]. Such actions might have led to the worsening of overall health, triggering infections with local and systemic complications, compromising general health, and forcing at risk patients to visit hospital emergency rooms [16, 24]. These complications were attributed to using the recommended 'advice, analgesics and antibiotics' (AAA) strategy to manage dental pain which has significant limitations with severe pain-related conditions, where face-to-face dental procedures are necessary to achieve symptomatic relief [25].

While TD offers acceptable reliability for the tentative diagnosis [26], the literature on tele-triage during the pandemic reveals knowledge gaps [16, 17, 21, 22, 27]. No previous research objectively reported whether tele-triage decisions were driven by the severity of pain. Likewise, to our knowledge, no studies compared the reported pain severity (PS) in relation to the associated symptoms and tentative tele-diagnosis. Studies have examined selective conditions e.g., dental-facial trauma [28], or pain, swelling and trauma [16], with less attention to other conditions associated with orofacial/dental pain e.g., loose/broken dental restorations, orthodontic appliances, oral ulcers, or bleeding. In addition, many reports focused on paediatric populations [28, 29], where PS cannot be objectively determined remotely.

Therefore, the current study assessed whether the severity of reported pain (exposure, continuous variables) affected the tele-triage decisions (binary outcome of whether the patient was managed remotely or referred for face-to-face management). PS was appraised across its associated tele-triage categories, dental history, and tentative tele-diagnosis to analyse their associations with tele-triage decisions. The emerging findings provide important information to a non-existent evidence base on the management of orofacial/dental pain during pandemics. The findings will provide practitioners and policy makers with information necessary to better manage reported orofacial/dental pain-related emergencies during situations like those experienced during the COVID-19 pandemic, or where resources are significantly limited for managing such emergencies.

## Methods

### Ethics, design and participants

The institutional research board (IRB) at Hamad Medical Corporation (HMC) granted permission for this service evaluation project to proceed. This is a retrospective analysis of information routinely collected for clinical audit and service evaluation. We analyzed patient and triage data of all hotline calls during the first wave of COVID-19 lockdown (N = 1239 across 5 months, 23 March–31 August 2020). We excluded callers with incomplete records where outcomes were not reported (n = 389), as well as children ≤ 8 years old (n = 125) as the numeric pain scale is not applicable to patients of this age [30, 31]. We also excluded patients who were not exposed to pain (0 pain score) (n = 193). The inclusion of those with no pain could introduce bias, as patients who did not report pain would be referred for face-to-face management for a reason other than the pain itself. After exclusions, the sample comprised 532 callers who reported various extents of pain.

We analyzed PS and its association with patient's age, sex, history of chronic illness, and dental discipline required. We also assessed the association between the PS and likelihood of the tele-triage decision of referral for face-to-face management, controlling for the same variables. Data were extracted from the electronic health records and the TD data collection form. The form was completed by each tele-dentist for every caller (Additional file 1: Fig. S1) [see Additional file 1], as part of an ongoing service audit.

### Setting and procedures

**Fig. 1:**
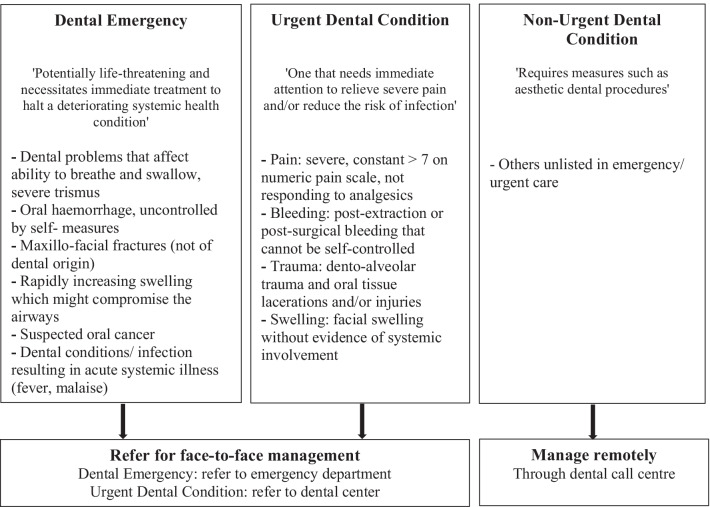
Recommended guidelines for tele-triage categorization and tele-triage decisions based on American Dental Association (ADA) [6], General Dental Council, UK [32], and Centers for Disease Control and Prevention (CDC) [33]

HDC set up a dental emergency services hotline managed by a team of qualified dentists to remotely consult, diagnose oral/ dental diseases, and undertake urgent tele-triage decisions. The TD policy and guidelines were formulated to guide the categorization of triage levels (emergency, urgent or non-urgent) and assist the team in the management of self-reported pain, swelling, bleeding, trauma, and oral-mucosal ulceration (Fig. 1).

These guidelines were not binding but rather indicative, leaving some freedom of interpretation by the tele-dentists, who were trained to ensure consistency of the service in arriving at triage and management decisions while observing caller privacy. The recommended policy and guidelines, based on American Dental Association (ADA) [6], General Dental Council, UK [32], and Centers for Disease Control and Prevention (CDC) [33], helped to triage the call, arrive at a tentative diagnosis, and accordingly offer appropriate care and/or referral. The guideline document was available to each practitioner and posted near the telephone set.

In addition, as part of the service, dentists administered a TD data collection form (Additional file 1: Fig. S1) [see Additional file 1] for each call. The form collected information on: 1) call (frequency, time, duration); 2) patient (demographics, medical/ allergy history, relationship of caller to patient, chief complaint, severity of pain on scale from 0 to 10); and, 3) triage outcomes (specialty required, management, referral to emergency/ dental facility undertaken, medications prescribed, procedure performed at point of referral).

### Pain

The numerical rating scale (NRS-11) is a pain assessment tool that is simple and easy to score [31], consisting of a total of 11 numbers, ranging from 0 to 10, representing no pain to the worst possible pain respectively [34]. NRS-11 is a reliable and valid self-report scale of pain intensity in many populations including children and adolescents as young as 8 years old; however, it is not suitable for very young children as certain cognitive skills are required for children to understand the meaning of the numbers of the NRS-11 and provide accurate PS ratings [35]. Hence, callers ≤ 8 years old were excluded.

### Outcome

Tele-dentists categorized the condition into emergency, urgent or non-urgent and managed the condition accordingly. AAA strategy was utilized as appropriate, and medications were home delivered to patients using courier service. Callers who were referred to a hospital emergency department or dental facility to receive face-to-face interventions were tele-triaged for COVID-19 symptoms and directed accordingly to either HDC or COVID-19 dedicated facilities. The focus of the current study is the association between pain severity and the triage decision of whether the patient was managed remotely or referred for face-to-face management.

### Statistical analysis

Descriptive and inferential statistics characterized the sample. Categorical variables are reported as frequencies and percentages. Differences in tele-triage decisions were compared using Fisher’s Exact test (Monte Carlo test with 99% CI) due to relatively small sample size. Continuous variables are reported as mean ± standard deviation, and PS was compared using t test when comparisons were between two groups or Analysis of variance (ANOVA) when > 2 groups. Binomial logistic regression assessed the association between the PS (exposure) as a continuous variable and likelihood of being referred for face-to-face management, adjusted for age, sex, history of chronic illness, and dental discipline needed.

In addition, sensitivity analysis was undertaken by including callers with pain score = 0 and comparing the emerging findings to those of the main analysis (excluding callers with pain score = 0) in order to explore the direction of any potential bias.

Dental discipline needed was included as a potential confounder in the regression analysis because some dental disciplines (e.g., restorative specialties including endodontics and prosthodontics) are known to be associated with procedures that generate considerable aerosols with a higher risk of transmission of the COVID-19 virus, [17], hence dental discipline could be factor associated with the outcome of whether the patient is referred or not. Also, in terms of the association of dental discipline with the exposure (pain), it is known that, on average, patients with endodontic pain for instance, report higher pain intensity than non-endodontic pain [36], while orthodontic pain varies during treatment [37]. Adjusted odds ratios are reported with their respective 95% CI. Statistical analysis was performed using IBM SPSS Statistics for Windows, version 25 (IBM Corp., USA). *P* < 0.05 (two-tailed) was considered statistically significant.

## Results

### Pain severity across selected sample characteristics

Table 1 illustrates that majority of callers were adults, while there was almost equal proportions of males and females. About three quarters of the sample had no history of chronic illness. More than half of the callers needed restorative specialties that operate mainly aerosol generating procedures.

**Table 1:** Pain severity across selected characteristics of the sample

Characteristic	N (%)	Pain Severity ^a^	*P-*value ^b^
Whole sample	532 (100)	6.05 ± 2.28	
Age group			< 0.0001
Child/ Adolescent 9–18 Y	69 (12.97)	4.91 ± 2.24	
Adult 19–44	365 (68.61)	6.29 ± 2.18	
Middle Aged 45–64	60 (11.28)	5.93 ± 2.35	
Aged 65 +	38 (7.14)	5.89 ± 2.63	
Gender			0.330
Male	252 (47.4)	6.15 ± 2.19	
Female	280 (52.6)	5.95 ± 2.36	
History of chronic illness			0.109
No	396 (74.44)	5.95 ± 2.28	
Yes	136 (25.56)	6.32 ± 2.26	
Dental discipline required			< 0.0001
Restorative Specialties	312 (58.65)	6.24 ± 2.23	
Surgical Specialities	150 (28.2)	6.23 ± 2.19	
Orthodontics	70 (13.16)	4.79 ± 2.31	

Pain severity (PS) represented as mean ± standard deviation; restorative specialties = restorative dentistry, endodontics & prosthodontics; surgical specialities = oral surgery & periodontics

^a^higher scores indicate higher severity of pain; ^b^ *P*-value based on ANOVA test for comparison of means

The mean PS for the whole sample was moderate/ high (6.05 ± 2.28), with adults reporting significantly more severe pain than other age groups. Callers who needed restorative and surgical specialties reported moderate/ high PS, while those who needed orthodontic procedures had significantly less PS. PS did not differ by caller sex or history of chronic illness.

### Association between pain severity and tele-triage decisions

Table 2 shows the regression analysis of the association between reported PS and tele-triage decision. Section A of the Table shows that an increase of 1 unit in pain score was significantly associated with 1.4 times increased odds of referral for face-to-face management. Three out of the remaining four variables included in the regression were also significantly associated with the tele-triage decision of referral for face-to-face management. The odds of face-to-face referral increased 2.1 times for pediatric/ adolescent patients (9–18 years). Likewise, those with history of chronic illness were 2.12 times more likely than those without to be referred for face-to-face management. Among the dental specialties, surgical and orthodontic specialties were each associated with increased odds of referral for face-to-face management (1.9 and 7.01 respectively) compared to restorative specialties. Sex was not significantly associated with referral.

**Table 2:** Regression analysis of the association between reported PS and tele-triage decision

	Tele-triage decision ^a^			
	Section A. Excluding patients reporting no pain* (N = 532)		Section B. Including patients reporting no pain* (N = 725)	
Variable	OR (95% CI)	*P* value	OR (95% CI)	*P* value
Pain Scale (per unit increase)	1.39 (1.26–1.54)	< 0.0001	1.29 (1.21–1.38)	< 0.0001
Age group^b^ (years)				
Adults (19–44)	1 (ref)		1 (ref)	
Child/ adolescent (9–18)	2.07 (1.07–4.02)	0.031	1.85 (1.09–3.13)	0.021
Middle aged (45–64)	1.73 (0.91–3.31)	0.096	1.89 (1.06–3.37)	0.030
Aged (65 +)	0.86 (0.36–2.05)	0.737	1.61 (0.80–3.24)	0.179
Gender				
Female	1 (ref)		1 (ref)	
Male	1.01 (0.67–1.51)	0.960	1.06 (0.74–1.52)	0.734
History of Chronic illness				
No	1 (ref)		1 (ref)	
Yes	2.12 (1.28–3.51)	0.003	1.66 (1.08–2.56)	0.021
Dental specialty needed				
Restorative specialties ^c^	1 (ref)		1 (ref)	
Surgical specialties ^d^	1.93 (1.22–3.04)	0.005	2.13 (1.40–3.25)	< 0.0001
Orthodontics	7.02 (3.54–13.87)	< 0.0001	4.86 (2.83–8.34)	< 0.0001

*OR* Adjusted odds ratios obtained for various factors using binomial regression, *CI* Confidence interval, *Ref* Reference

^a^ outcome categorized as follow; 0 = Remote management, 1 = referral for face-to-face management; ^*b*^age groups categorized as standard age ranges defined by the Medical Subject Headings (MeSH); ^c^ restorative specialties = restorative, endodontics and prosthodontics; ^d^ surgical specialties = oral surgery and periodontics

* no pain = 0 score on reported pain scale

Section B of the Table 2 shows that including pain score = 0 generally did not influence the findings except in two instances: it resulted in a lower likelihood of referral for those requiring orthodontic care, and a higher likelihood of referral of older patients.

We repeated the regression with and without adjusting for dental discipline. Not adjusting for discipline increased the significance and effect size of the child/ adolescent age group (Additional file 2: Table S1) [see Additional file 2], probably because most of them required orthodontic treatment and were referred accordingly. This suggested that it was appropriate to adjust for dental discipline.

### Tele-triage categories, tele-triage decisions, and their relation to PS

Table 3 shows that PS differed significantly across the different triage categories, and that the emergency category had the highest PS. Likewise, tele-triage decision significantly differed among different triage categories, were for all calls triaged as emergency, the tele-triage decision was to refer for face-to-face management; most of calls triaged as urgent were referred; whilst very few of calls triaged as not urgent were referred. Although management decisions were based on the triage categories, however, when the PS was tested against the management decisions undertaken, there were also significant differences between them. Calls that were remotely managed were found to have a significantly lower PS than those where the management decision was to refer.

**Table 3:** Tele-triage categories, tele-triage decisions, and their relation to pain severity

	Tele-triage decision ^b^		Total
	Remote management N (%)	Referal N (%)	N (%)
*Triage categories*			
Emergency (*PS* 8.00 ± 2.83)^a^	0 (0)	4 (100)	4 (100)
Urgent (*PS* 7.13 ± 2.05)^a^	31 (16.6)	156 (83.4)	187 (100)
Not urgent (*PS* 5.43 ± 2.17) ^a^	331 (97.1)	10 (2.9)	341 (100)
Total	362 (68.07)	170 (31.95)	532 (100)
Pain severity	5.69 ± 2.23	6.79 ± 2.23	

*PS* Pain severity on the numerical rating scale (NRS-11); all percentages represent row percentages

^a^ differences in PS across different triage categories and PS (ANOVA), *P* < 0.0001; ^b^ differences in tele-triage decisions across triage categories (Fischer’s exact test), *P* < 0.0001; differences in PS among different tele-triage decisions (ANOVA), *P* < 0.0001

### PS differences across associated dental history, tentative diagnosis, and tele-triage decisions

In terms of dental history (Table 4), although tooth with cavity or filling were the most frequent and most painful, only 21.78% were referred for face-to-face management. Similarly, jaw-related symptoms had the second highest PS, but only 40% of those callers were referred. Conversely, while orthodontic appliance problems had the lowest PS, 60% of these problems were referred for face-to-face clinical interventions.

**Table 4:** Pain severity differences across diverse dental histories, tentative diagnosis, and teletriage decisions

Characteristic	N (%)	Pain severity^*a*^	*P*-value*	Decision		*P*-value**
				remote management^*b*^	Referal	
Dental history			< 0.0001			< 0.0001
Tooth with cavity or filling	202 (37.97)	6.65 ± 2.02		158 (78.22)	44 (21.78)	
Jaw symptoms	10 (1.88)	6.60 ± 2.54		6 (60.0)	4 (40.0)	
Wisdom tooth	43 (8.08)	6.35 ± 2.20		33 (76.74)	10 (23.26)	
Swelling	86 (16.17)	6.17 ± 2.18		54 (62.79)	32 (37.21)	
Recent tooth extraction	4 (0.75)	5.75 ± 3.40		3 (75.0)	1 (25.0)	
Dental restoration/ prosthesis	39 (7.33)	5.74 ± 2.67		24 (61.54)	15 (38.46)	
Dental trauma or tooth fracture	40 (7.52)	5.70 ± 2.11		25 (62.5)	16 (37.5)	
Bleeding	13 (2.44)	5.23 ± 2.31		12 (92.31)	1 (7.69)	
Ulcer	13 (2.44)	5.23 ± 2.45		8 (61.54)	5 (38.46)	
Inflamed gums	14 (2.63)	5.07 ± 1.94		13 (92.86)	1 (7.14)	
Orthodontic appliance	68 (12.78)	4.71 ± 2.35		27 (39.71)	41 (60.29)	
Tentative diagnosis			< 0.0001			< 0.0001
Cellulitis	4 (0.75)	7.75 ± 2.87		0 (0.0)	4 (100)	
TMD	8 (1.50)	6.75 ± 2.37		4 (50.0)	4 (50.0)	
Pulpitis	169 (31.77)	6.74 ± 2.07		129 (76.33)	40 (23.67)	
Dental caries	52 (9.77)	6.63 ± 1.78		40 (76.92)	12 (23.08)	
Pericoronitis	57 (10.71)	6.25 ± 2.21		44 (77.19)	13 (22.81)	
Dry socket	5 (0.94)	6.20 ± 3.11		3 (60.0)	2 (40.0)	
Dental abscess	46 (8.65)	6.11 ± 2.04		31 (67.39)	15 (32.61)	
Dental fracture	39 (7.33)	5.77 ± 2.24		23 (58.97)	16 (41.03)	
Sialadenitis	2 (0.38)	5.50 ± 2.12		0 (0.0)	2 (100)	
Broken dental filling/ prosthesis	32 (6.02)	5.38 ± 2.69		19 (59.38)	13 (40.62)	
Ulcer	13 (2.44)	5.23 ± 2.45		8 (61.54)	5 (38.46)	
Periodontitis	35 (6.58)	4.97 ± 2.05		32 (91.42)	3 (8.58)	
Broken orthodontic appliance	70 (13.16)	4.70 ± 2.31		29 (41.43)	41 (58.57)	

*TMD* Temporomandibular dysfunction

* *P* value based on ANOVA test for comparison of means; ** *P* value based on Fisher’s Exact (Monte Carlo test with 99% CI)

^*a*^ M ± SD, higher scores (1–10) indicate higher severity of pain, ^*b*^ comprising AAA: advise, analgesics antibiotics

As for the tentative tele-diagnoses (Table 4), cellulitis was associated with the highest pain, and all cases were referred to a hospital emergency facility. Temporomandibular dysfunction (TMD) was also associated with high pain levels and 50% were referred for clinical management. Pulpitis was the most common tele-diagnosis (31.77%) and was significantly associated with high PS, however, about three quarters of these patients were managed remotely. While orthodontic problems were associated with the lowest pain levels, more than half of the callers could not be managed remotely and were referred for chairside interventions.

## Discussion

There has been calls to enhance the evidence-base of the management of orofacial/ dental pain [38]. The main objective of the current study was to explore whether PS was associated with tele-triage decisions (remote management of caller vs referral for face-to-face management). To our knowledge, no previous research undertook such task. Hence, it was not straightforward to directly compare our findings with previous research. The main finding was that tele-triage decision to remotely manage or to refer the caller for face-to-face management was significantly associated with the severity of the orofacial/ dental pain. An increase in the pain score was associated with increased odds of face-face referral, however, other covariates were also associated with such decisions. The most significant associations were whether the caller had a history of a chronic illness, and whether the discipline that was needed operated aerosol generating procedures. This indicates that management decisions at HDC were highly individualized, taking into account the tripartite considerations of the reported pain, clinical judgement, and the high transmission characteristics of COVID-19.

For instance, for callers with high PS, in terms of dental history, those with problems related to tooth cavity/filling, temporo-mandibular joint, wisdom tooth, and swellings had the highest PS. However, more than two thirds of these patients were managed remotely. In terms of tele-diagnosis, we observed that pulpitis was not only associated with high PS, but it was also the most frequent tele-diagnosis (31.77%), concurring with the range of 22.5–46% pulpitis rates reported by other studies as the origin of orofacial/ dental pain during the pandemic [16, 19, 20]. Despite that endodontic root canal treatment (RCT) is the gold standard treatment for pulpitis, > 75% of our pulpitis were managed remotely, because RCT generates aerosols. Hence RCT was restricted during the pandemic and reserved for when the tooth was restorable and strategically important, otherwise extraction was favoured. This supports a UK study where 65% of clinical consultations resulted in extractions to avoid aerosol generating procedures [17]. This also explains the increased odds of the patients being seen at the clinic for surgical and orthodontic procedures (OR = 1.9 and 7.016 respectively) compared to that of aerosol generating restorative procedures.

Hight levels of PS were observed with conditions that could precede a serious medical emergency or are caused by odontogenic infections. Our management decisions considered the risk of systemic life-threatening complications in tandem with PS, as recommended by the ADA [6]. For instance, we observed that TMD was the second most painful condition. This concurs with that the pandemic adversely effected peoples' psycho-emotional status and intensified their bruxism and TMD symptoms [39]. However, during tele-triage, patients may provide inaccurate history [40] as TMD is difficult to be described by the patient. In addition, it could be an early symptom of an underlying cardiac problem [41]. Given such uncertainties combined with the fact that our TD was largely limited to telephone consultations during the pandemic's initial stages like other places worldwide [39], half of our TMD cases were referred. Likewise, although we found that cellulitis had the highest PS, pain was not the sole reason for referral. It is recognized that cellulitis of odontogenic infection origins can be life threatening [42]. All cellulitis were categorized by the HDC tele-dentists as emergency and referred to hospital emergency for intravenous antimicrobials and analgesics [42].

For callers with low PS, the individualized approach after careful weighting of the reported pain and clinical picture was also evident. For instance, those with history of orthodontic appliance problems experienced the least pain, but the majority were referred. The majority of orthodontic patients typically fell in the pediatric/ adolescent group (9–18 years), and this could explain the 2.1 times increased odds of face-to-face referral for patients of this age group. Orthodontic appliance related problems may not be life threatening or severely painful; nevertheless, they challenge daily activities (speech, mastication, sleep), quality of life, and could lead to significant consequences if left unattended.

At the height of a rapidly changing pandemic, HDC management decisions provide positive lessons for future 'normal' times after the pandemic. On the one hand, during the pandemic, severe orofacial/ dental pain was the driver of urgent care [22]. On the other hand, pre-pandemic literature suggests that the high orofacial/ dental pain results in an elevated demand of emergency appointments at dental practices, some general medical practices and emergency departments [14]. The present study showed that PS indeed influenced the tele-triage decisions of orofacial/ dental pain (remote management or referal). However, a main observation is that not all our cases with higher severity of pain were referred; and conversely, not all cases with low pain severity were managed remotely. Such discrepancies might seem odd. However, when dental history, tentative tele-diagnosis, discipline required and face-to-face procedure at point of referral were considered, other factors like clinical judgment, risk of disease transmission, potential life-threatening systemic complications, possible diagnostic uncertainty, and probable challenges with daily activities/ deteriorated quality of life if the condition is left unattended contribute to explain such discrepancies. The approach used at HDC during the pandemic led to the remote management of 31 cases triaged as urgent (5.8% of the total callers) and 331 cases triaged as non-urgent (62.2% of the total callers). During 'normal' (non-pandemic) times, these combined 68% of the patients would have otherwise physically presented themselves at our clinics.

The current study has limitations. HDC is a tertiary dental services provider, and patients in the current study probably had previous dental experiences or persisting symptoms. Such patients are different than those who present at primary dental care centers experiencing their first dental pain encounter. The current cross sectional design does not allow any inferences of causal relationships between pain severity and triage decision decisions, rather only associations and should be interpreted as such. We encountered 40% missing data regarding the outcome during the early phase of the study when the dentists were home-working during the lockdown. This influenced the effective sample size used in the analysis. Others have similarly reported that the 'helpline was developed and implemented while many people were working from home during shelter-in-place restrictions, making training, calibration on information entry, and supervision more difficult. Despite conducting training sessions, initially there was inconsistent or incomplete recording of information in the spreadsheet for administrative calls and in the newly adopted Epic' [21, p. 833]. For the current study as well as for other studies, such missing data could influence the robustness of the findings as it could over or under estimate the findings. The confidence intervals we observed were rather wide in some instances e.g., for the odds ratio of the orthodontics category of the dental specialty needed (Table 2); larger sample size would have been useful. Hence, generalizability of the findings needs to exercise caution, and future research would benefit to address these points. Despite this, the study has strengths. Previous work on patient-reported orofacial/ dental pain during the pandemic lockdown focused on the association between worsened socioeconomic conditions and pain severity [43], while the current study explored the influence of PS on tele-triage decisions, and appraised PS across triage categories, demographic variables, dental history, discipline required, and tele-diagnosis related factors. These findings provide policy makers with information necessary to better manage orofacial/ dental pain related emergencies and similar epi/pandemics, or where resources are significantly limited in rural areas and underdeveloped regions.

The main public health implication of this study is that extending the application of teledentistry, tele-triage, tele-consultation, and tele-therapy to after the pandemic is over might offer a promising and useful approach if practiced with the same level of individualization and driven by considerations of both, the reported pain and clinical judgement. Such continuation of tele-services after the pandemic would lead to the freeing of healthcare resources that could be used elsewhere, shortening of long waiting lists, alleviation of over-crowded walk-in clinics, and reserving the chairside appointments to conditions that truly require such management.

Future research should aim at larger sample sizes and less missing values. There is also a need for evidence premised on different demographic profiles and countries to better define dental characteristics that fall in the basic, urgent, or emergency categories of essential oral health care and the best route of action, in terms of remote or face-to-face management for each of these categories.

## Conclusion

During this phase of the pandemic, the most frequent symptom with most severe orofacial/ dental pain was decayed or filled tooth, and the most frequent tele-diagnosis was pulpitis. PS was significantly higher for emergency and urgent tele-triage categorizations. Tele-triage decisions were significantly influenced by severity of patient-reported orofacial/ dental pain, adjusting for a range of variables. However, acute dental conditions were not inevitably referred when they were judged to be manageable remotely; and conversely, less acute dental conditions were given the attention they required and referred when they were gauged to have potential life threating or quality of life implications. Such measured actions could lead to sizeable savings of healthcare resources which could, in future, be used to reduce pressure on clinics overcrowded with conditions that could be remotely managed. Hence, the continuation of such tele-approach later after the pandemic might be safe and useful for a range of scenarios and healthcare facilities if practiced with similar level of individualization and driven by careful considerations of the combination of reported pain and complaints on the one hand, and the meticulous clinical judgement on the other.

## Supplementary Information

**Additional file 1**.** Fig. S1**: Teledentistry data collection form

**Additional file 2**.** Table S1**: Regression analysis with and without adjusting for dental discipline required

## Data Availability

The datasets generated and/or analysed during the current study are not publicly available due to the institution regulations, however, they are available from the corresponding author on reasonable request.

## Abbreviations

COVID-19: Coronavirus disease 2019
WHO: World Health Organization
CDC: Centers for Disease Control and Prevention
HDC: Hamad dental center
TD: Teledentistry
ADA: American Dental Association
PS: Pain severity
AAA: Advise, analgesics and antibiotics
IRB: Institutional research board
NRS-11: Numerical rating scale
ANOVA: Analysis of variance
RCT: Root canal treatment
OR: Adjusted odds ratios obtained for various factors using binomial regression
CI: Confidence interval
Ref: Reference
