# Supplementary material for: Patient-reported orofacial-dental pain severity and tele-triage decisions during COVID-19 pandemic: Does the severity of pain drive tele-triage decisions?
Source: BMC Oral Health. 2022 Jul 27;22:310. doi: 10.1186/s12903-022-02340-w (PMC9326137; doi:10.1186/s12903-022-02340-w)
Supplement: Supplementary file 1 — Additional file 1. Fig. S1: Teledentistry data collection form [file 12903_2022_2340_MOESM1_ESM.docx]

| Patient electronic health record number |  | | | | | | | | | | | | | | | |
| --- | --- | --- | --- | --- | --- | --- | --- | --- | --- | --- | --- | --- | --- | --- | --- | --- |
| Date of call |  | | | | | | | | | | | | | | | |
| Time of call | AM | | | | | | | PM | | | | | | | | |
| Duration of call |  | | | | | | | | | | | | | | | |
| Nationality |  | | | | | | | | | | | | | | | |
| Age |  | | | | | | | | | | | | | | | |
| Gender | Male | | | | | | | Female | | | | | | | | |
| Previous medical history |  | | | | | | | | | | | | | | | |
| History of allergies |  | | | | | | | | | | | | | | | |
| Patient calling by himself | Yes | | | | | | | | | | | | | | | |
|  | No, Caller-Patient Relationship | | | | | | | | | | | | | | | |
|  | Father | | | | | Mother | | | | | | | | Son | | |
|  | Daughter | | | | | Care Giver | | | | | | | | Other: | | |
| Chief complaint | Pain | | Bleeding | | | Trauma | | | | Swelling | | | | Ulcer | | Other |
|  | Explain other: | | | | | | | | | | | | | | | |
| Pain Scale | 0 | 1 | | 2 | 3 | | 4 | | 5 | | 6 | 7 | 8 | 9 | 10 | |
| Triage Category | Emergency | | | | | Urgent | | | | | | | | Non-urgent | | |
| Dental discipline required | Orthodontics | | | | | Endodontics | | | | | | | | Prosthodontics | | |
|  | Periodontics | | | | | Oral Surgery | | | | | | | | Pedodontics | | |
| Management decision | Refer to nearest accident and emergency facility | | | | | | | | | | | | | | | |
|  | Refer to nearest dental facility offering urgent dental care | | | | | | | | | | | | | | | |
|  | Instructions only | | | | | | | | | | | | | | | |
|  | Instructions and medications | | | | | | | | | | | | | | | |
| Medications prescribed | No | | | | | Analgesics | | | | | | | | Mouth wash | | |
|  | Antibiotics | | | | | Topical | | | | | | | | Other: | | |

**Fig. S1.** Teledentistry data collection form.
