# Supplementary material for: Patient-reported orofacial-dental pain severity and tele-triage decisions during COVID-19 pandemic: Does the severity of pain drive tele-triage decisions?
Source: BMC Oral Health. 2022 Jul 27;22:310. doi: 10.1186/s12903-022-02340-w (PMC9326137; doi:10.1186/s12903-022-02340-w)
Supplement: Supplementary file 2 — Additional file 2. Table S1: Regression analysis with and without adjusting for dental discipline required [file 12903_2022_2340_MOESM2_ESM.docx]

|  | Teletriage decision ^a^ (N=532) | | | |
| --- | --- | --- | --- | --- |
|  | Adjusting for dental discipline required | | Not Adjusting for dental discipline required | |
| Variable | OR (95% CI) | *P* value | OR (95% CI) | *P* value |
| **Pain Scale (per unit increase)** | 1.39 (1.26 - 1.54) | < 0.0001 | 1.31 (1.19-1.44) | < 0.0001 |
| **Age group ^b^ (years)** |  |  |  |  |
| Adults (19-44) | 1 (ref) |  | 1 (ref) |  |
| Child/ adolescent (9-18) | 2.07 (1.07- 4.02) | .031 | 3.84 (2.15-6.88) | < 0.0001 |
| Middle aged (45-64) | 1.73 (0.91 - 3.31) | .096 | 1.50 (0.81-2.79) | .199 |
| Aged (65+) | 0.86 (0.36 - 2.05) | .737 | 0.77 (0.34-1.79) | .553 |
| **Gender** |  |  |  |  |
| Female | 1 (ref) |  | 1 (ref) |  |
| Male | 1.01 (0.67 - 1.51) | .960 | 0.87 (0.59-1.28) | .488 |
| **Chronic illness** |  |  |  |  |
| No | 1 (ref) |  | 1 (ref) |  |
| Yes | 2.12 (1.28 -3.51) | .003 | 2.16 (1.34-3.50) | 002 |
| **Dental Specialty Needed** |  |  |  |  |
| Restorative specialties ^c^ | 1 (ref) |  |  |  |
| Surgical specialties ^d^ | 1.93 (1.22 - 3.04) | .005 |  |  |
| Orthodontics | 7.02 (3.54 -13.87) | < 0.0001 |  |  |

Table S1. Regression analysis with and without adjusting for dental discipline required.

^a^ outcome categorized as follow; 0=Remote management, 1= referral for face-to-face management; *^b^* age groups categorized as standard age ranges defined by the Medical Subject Headings (MeSH); ^c^ restorative specialties = restorative, endodontics and prosthodontics; ^d^ surgical specialties = oral surgery and periodontics; OR = Adjusted odds ratios obtained for various factors using binomial regression; CI = confidence interval; Ref = reference.
